# Factors Affecting the Potential Efficacy of Intrauterine Platelet-Rich Plasma Infusion on Thin Endometrium in Women with Recurrent Implantation Failure

**DOI:** 10.3390/jpm13091419

**Published:** 2023-09-21

**Authors:** Pin-Yao Lin, Chun-I Lee, Yi-Chun Chen, En-Hui Cheng, Chun-Chia Huang, Chung-I Chen, Tsung-Hsien Lee, Yu-Jen Lee, Maw-Sheng Lee

**Affiliations:** 1 Institute of Medicine, Chung Shan Medical University, No. 110, Sec. 1, Jianguo N. Rd., South District, Taichung City 40201, Taiwan; jellylin0607@gmail.com (P.-Y.L.);; 2 Division of Infertility, Lee Women’s Hospital, No. 30-6, Sec. 1, Changping Road, Beitun District, Taichung City 406, Taiwanyujen1027@gmail.com (Y.-J.L.); 3 Department of Obstetrics and Gynecology, Chung Shan Medical University Hospital, No. 110, Sec. 1, Jianguo N. Rd., South District, Taichung City 40201, Taiwan

**Keywords:** thin endometrium, platelet lysate, platelet-rich plasma, recurrent implantation failure, frozen embryo transfer, endometrial receptivity, endometrial injury

## Abstract

Optimizing endometrial thickness (EMT) is crucial for successful embryo implantation, but enhancing thin endometrium remains a significant challenge. Platelet-rich plasma (PRP)-derived therapies have emerged as a promising approach in reproductive medicine due to their capacity to facilitate tissue repair and regeneration. This study aims to identify the risk factors associated with the failure of intrauterine PRP infusion for thin endometrium in women with recurrent implantation failure (RIF). We retrospectively reviewed data from 77 women with RIF, all exhibiting an EMT of <7 mm. These women underwent programmed hormone therapy for frozen embryo transfer (FET) and received two autologous intrauterine PRP infusions. Following intrauterine PRP-lysate (PL) infusions, the mean increase in EMT was 1.9 ± 1.2 mm, with EMT reaching 7 mm in 86% of the cases (66/77; average EMT, 8.3 mm). We identified an exceedingly thin EMT as a risk factor impacting the therapeutic efficacy in increasing EMT (*p* = 0.04, OR: 3.16; 95% CI: 1.03–9.67). Additionally, the number of previous uterine surgeries emerged as a prognostic factor for pregnancy failure following PL infusion (*p* = 0.02, OR: 2.02; 95% CI: 1.12–3.64). Our findings suggest that an extremely thin EMT and a history of numerous uterine surgeries can impede successful pregnancy, even when an optimal EMT is achieved following PRP infusion.

## 1. Introduction

Optimal endometrial thickness (EMT) plays a critical role in embryo implantation. Numerous studies have underscored a positive correlation between EMT and embryo implantation rates, citing a cut-off value of 7 mm as an indication for lower clinical pregnancy likelihood [1]. Consequently, this has established a clinical precedent to defer embryo transfer when the patient’s EMT measures below this threshold. A thin endometrium is not only implicated in recurrent implantation failure (RIF) [2], but it also adversely influences obstetric outcomes [3,4,5].

Several strategies have been recommended to ameliorate thin EMT; however, an effective therapeutic solution remains elusive [6,7]. Autologous platelet-rich plasma (PRP), a fraction of plasma with a heightened concentration of platelets [8], has garnered attention in reproductive medicine due to its high concentration of bioactive substances such as growth factors and cytokines [9]. Intrauterine infusion of PRP enables these platelet-stored factors to interact with the endometrium, promoting cell proliferation, neoangiogenesis, and delivering anti-inflammatory effects, thereby facilitating successful implantation [10].

Chang et al. pioneered the application of PRP in treating human thin endometrium, concluding that intrauterine PRP infusion augments endometrial growth and enhances pregnancy outcomes [11]. A multitude of subsequent studies have supported the use of PRP therapy [12,13,14,15,16,17,18,19,20,21,22]. Recent literature reviews demonstrated that intrauterine PRP infusion enhances endometrial thickness, implantation rates, clinical pregnancy rates, and live birth rates in women with previous implantation failure. However, the confirmation of its efficacy necessitates additional high-quality studies [16,17,23,24]. Notwithstanding, the current literature displays a lack of uniformity in terms of indications, inclusion criteria, and protocols, resulting in inconsistent outcomes and disparate findings. To our knowledge, an exhaustive investigation into risk factors predictive of intrauterine PRP infusion failure for thin endometrium is lacking. The purpose of this retrospective study is to identify the risk factors predictive of PRP failure in RIF cases with thin endometrium.

## 2. Materials and Methods

### 2.1. Selection of Patients

This investigation utilized a retrospective design, carried out at Lee Women’s Hospital from March 2018 to August 2019. The causes of infertility in the study population are male factor (n = 4; 5.1%), ovarian factor (n = 20; 6%), tubal factor (n = 3; 3.9%), and mixed factor (n = 50; 65%). The study obtained approval from Chung Shan Medical University Hospital’s Institutional Review Board (IRB approval no. CS18028). Following informed consent, 77 women undergoing IVF presenting with thin endometrium were enrolled. Inclusion criteria entailed the following: (1) RIF, indicated by unsuccessful implantation after four cumulative transfers of good-quality cleavage-stage embryos or blastocysts (maximum of four for cleavage embryo and two for blastocyst per transfer) within two fresh or frozen cycles [25]; (2) EMT of less than 7 mm on days 11–13 of the menstrual cycle following standard hormone therapy for FET; (3) absence of significant intrauterine adhesions as evidenced by hysteroscopy within the preceding six months. Exclusion criteria encompassed the following: (1) active anticoagulant treatment; (2) hematologic disorders (platelet count less than 150 × 10^3^/µL or hemoglobin below 11 g/dL); (3) body mass index (BMI) equal to or exceeding 30 kg/m^2^; (4) severe conditions such as endometriosis, cancer, or other diseases. Detailed records of any prior uterine surgery potentially linked to endometrial damage, such as uterine curettage or dilation, hysteroscopic polypectomy, adhesiolysis, myomectomy/adenomyomectomy, or operations for congenital uterine anomalies, were meticulously maintained.

### 2.2. Autologous PRP-Lysate Preparation

The production of PRP-lysate (PL) involves the mechanical disruption of platelets within a platelet-rich concentrate, yielding a range of activated growth factors [26]. In comparison to traditional PRP, PL is a cell-free supernatant utilized to supplement mesenchymal stem cell cultures [27], promote wound healing [28], and as an innovative therapy in regenerative medicine [29]. Blood samples of 20 mL were collected from each patient and drawn into two Acti-PRP tubes (Aeon Biotherapeutics Corp., Taipei, Taiwan) without anticoagulant using a sterile syringe. These tubes were then subjected to centrifugation at 3600 rpm for six minutes at room temperature (A500, Aeon Biotherapeutics Corp., Taipei, Taiwan). Subsequent to centrifugation, 0.5 mL of the resultant yellow plasma was carefully removed, ensuring approximately 0.5 mL of plasma remained in each tube. The tubes were then inverted at least 30 times to facilitate plasma–buffy coat interaction. Following solidification of the PRP concentrate, mechanical disruption was achieved via gentle stirring with a needle until the formation of a gelatinous mass was achieved, a step which was repeated three to four times to ensure complete clot inhibition. Ultimately, 1 mL of PL concentrate was extracted using a sterile syringe, and promptly infused into the uterine cavity within 30 min of collection.

### 2.3. Quantification of TGF-β1, PDGF-BB, PDGF-AB, VEGF, and VEGF-A in Blood and PL

PL was analyzed to determine the concentrations of TGF-β1, PDGF-BB, PDGF-AB, VEGF, and VEGF-A, which are implicated in endometrial development and receptivity [30,31,32]. These growth factors were quantified in accordance with their respective ELISA kit protocols. The human TGF-β1 ELISA kit (ab100647, Abcam, Cambridge, UK), human PDGF-BB ELISA kit (ab184860, Abcam, Cambridge, UK), human PDGF-AB ELISA kit (ab100623, Abcam, Cambridge, UK), human VEGF ELISA kit (ab222510, Abcam, Cambridge, UK), and human VEGF-A ELISA kit (ab119566, Abcam, Cambridge, UK) were employed for the respective quantification of TGF-β1, PDGF-BB, PDGF-AB, VEGF, and VEGF-A.

### 2.4. Endometrial Preparation, PL Infusion, and ET

Patient endometria were prepared for FET via a programmed hormone therapy regimen as delineated in prior work [33]. This preparation commenced on the third day of the menstrual cycle, with patients receiving daily oral estradiol valerate (Estrade, Synmosa, Taipei, Taiwan). EMT measurements were taken on the 11th to 13th day of the cycle, utilizing transvaginal ultrasound conducted in the mid-sagittal plane. EMT was assessed independently by three experienced sonographers, measuring the thickest echogenic area from one stratum basalis endometrial interface to the other [34]. Additionally, the pulsatility index (PI) and resistive index (RI) of uterine arteries at the cervico-corporeal level were measured [35]. Patients with an EMT < 7 mm, who fulfilled the predetermined inclusion criteria, underwent intrauterine autologous PL infusion twice at a 48 h interval. Post-second infusion, EMT was reevaluated three days later. If EMT remained <7 mm, the ET was discontinued. The mean duration from the start of endometrial preparation on Day 3 until progestin commencement was 17.2 days. However, if the EMT measured ≥7 mm, ET proceeded on Day 3 following 3.5 days of progestin use or on Day 5 following five days of progestin use [33]. Of the total patients, 13 underwent Day 3 ET and the remaining 53 underwent Day 5 ET.

### 2.5. Study Outcomes

The primary objective of this study was to elucidate the determinants affecting the successful attainment of an EMT ≥ 7 mm following PRP infusion. The secondary objective aimed to identify factors influencing the pregnancy rate following FET. The outcome measures included the clinical pregnancy rate (CPR), implantation rate (IR), miscarriage rate (MR), and live birth rate (LBR). The IR was calculated as the ratio of gestational sacs to the total number of transferred blastocysts. Clinical pregnancy was defined as the presence of an intrauterine gestational sac along with a positive cardiac activity observed on ultrasound during the 6–8-week gestational period [36]. The MR was determined as the fraction of confirmed spontaneous intrauterine pregnancy losses from all clinical pregnancies, confirmed via ultrasound [37]. The LBR was defined as the birth of at least one live-born child. The cumulative pregnancy rate was traced until July 2020.

### 2.6. Statistical Analysis

Differences in EMT pre- and post-PRP infusion were analyzed using the Wilcoxon signed-rank test. The binomial logistic regression model was employed to scrutinize the correlation between patient characteristics and PRP infusion outcomes. The Mann–Whitney U test was utilized to evaluate the average number of preceding endometrial injuries between the group that underwent ET (ET group) and the group in which ET was canceled (ET-canceled group). The parameters of IVF-ET were analyzed using both univariate and multivariate analyses to identify predictive risk factors for PRP failure. All data were analyzed using SPSS (version 20.0; IBM Corporation, Armonk, NY, USA), with a *p*-value < 0.05 considered significant. The “R 4.2.0” statistical software, along with the “ggplot2” and “ggpubr” packages, were used for data visualization and statistical correlation analysis between PRP components and pregnancy.

## 3. Results

### 3.1. General Characteristics of the Study Population

A total of 77 RIF women with EMT < 7 mm who underwent programmed hormone therapy for FET were enrolled in this study (Table 1). The mean age of the participants was 38.6 ± 6.5 years, with a median infertility duration of 2.8 years. Secondary infertility represented 73% of the cases. Participants had a median of three unsuccessful IVF cycles and a mean of 1.7 prior uterine surgeries.

### 3.2. Clinical Outcomes following PL Infusion

Upon performing the intrauterine PL infusion twice, 86% (n = 66) of the participants achieved an EMT of 7 mm or greater, allowing for ET. Conversely, in 11 cases, ET was canceled due to an EMT of less than 7 mm. Thus, in 14% of the cases, PL infusion did not result in an optimal EMT increase. Correlations were identified between the therapeutic effect post-PL infusion and the number of previous failed IVF cycles (*p* = 0.011) and the initial EMT prior to PRP treatment (*p* = 0.014). A trend was also noted showing a negative correlation between the number of past uterine surgeries and the increase in EMT (Table 2).

The procedure was well tolerated without severe adverse events, and no participants dropped out. In the ET group, EMT increased significantly by 29.7% (from 6.4 mm to 8.3 mm), but only by 7.1% (from 5.6 mm to 6.0 mm) in the ET-canceled group. The IR, CPR, MR, and LBR in the ET group were 21% (25/119), 33% (22/66), 27.3% (6/22), and 21% (14/66), respectively. Notably, the pregnancy rate following PL infusion was negatively correlated with the number of prior uterine surgeries (*p* < 0.001) (Table 3).

### 3.3. Factors Affecting EMT Increase and Pregnancy Outcomes after PL Infusion and Growth Factor Level

**Figure 1:**
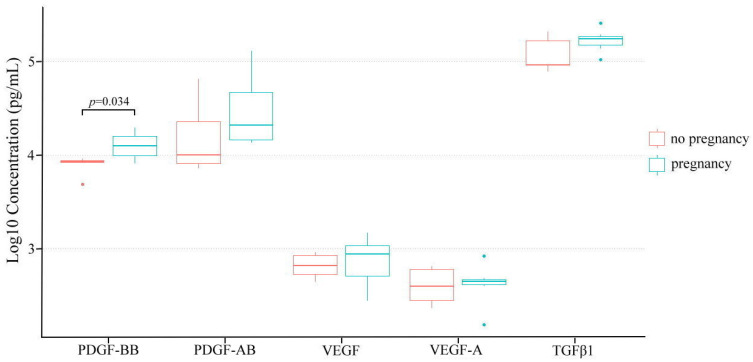
Comparison of selected growth factors in PL between pregnancy and non-pregnancy groups. Each box in the figure represents a group’s distribution of values: the horizontal lines within denote median values; the boxes extend from the 25th to the 75th percentiles; the vertical lines extending from the boxes represent adjacent values (i.e., the most extreme values within 1.5 interquartile range of the lower and upper quartiles); outliers are denoted by dots. The *p*-value, calculated using Student’s *t*-test, indicates the statistical significance of the differences observed.

Both univariate and multivariate logistic regression models identified pre-infusion EMT as a significant risk factor impacting EMT increase (*p* = 0.004, OR: 3.16; 95% CI: 1.03–9.67) (Table 4). The number of previous uterine surgeries was confirmed as a prognostic factor linked to pregnancy failure following PL infusion in the multivariate analysis (*p* = 0.02, OR: 2.02; 95% CI: 1.12–3.64). An analysis of selected growth factors in the pregnant and non-pregnant groups revealed a significant difference in the concentration of PDGF-BB between the two groups (7883.94 ± 1712.14 vs. 13,143.29 ± 4500.70, *p* = 0.048). No significant differences were observed in other selected growth factors (Figure 1).

## 4. Discussion

The purpose of this study was to determine the risk factors predictive of EMT increase and subsequent pregnancy failure following intrauterine PL infusion. Our findings showed a mean EMT increase of 1.9 mm and the achievement of EMT > 7 mm in 86% of instances. PL, akin to other PRP products, seemed to effectively increase EMT. A CPR of 33% and an LBR of 21% were observed in RIF patients with thin endometrium, suggesting potential advantageous effects of autologous PL on endometrial receptivity. It was demonstrated in this study that pre-infusion EMT impacted the increase in EMT. Additionally, it was found that the number of previous uterine surgeries was a significant risk factor for pregnancy failure following PL infusion. The level of PDGF-BB in PL was notably higher in the pregnant cohort.

Numerous proposed mechanisms exist to elucidate the adverse effects of thin endometrium on reproductive outcomes, including abnormalities or dysfunction in the estrogen receptor [38], excess oxygen tension in the thin endometrial environment [5], impaired angiogenesis [39], abnormal immunology, and diminished response to oxidative stress [40]. PRP, being enriched with growth factors and various biologically active substances, is hypothesized to ameliorate EMT. Several investigations have reinforced the initial report by Chang et al. [11] suggesting that PRP infusion might stimulate endometrial growth and enhance pregnancy outcomes [12,13,14,41,42,43]. A recent systematic review and meta-analysis [23] proposed that PRP could serve as an alternate therapeutic approach for patients with thin endometrium and RIF. However, there is still a paucity of evidence for the use of PRP in the management of thin endometrium to improve pregnancy outcomes. Some limitations of previous studies include inadequate study design, small sample sizes with clinically irrelevant endpoints, and a lack of detailed PRP preparation information [44]. An additional significant confounding factor is the considerable heterogeneity among study populations, including patients with thin endometrium with or without failed ET, RIF patients with or without thin endometrium, and patients with intrauterine adhesions.

Furthermore, the suitability of patients with a thin EMT for intrauterine PRP therapy remains unknown. Within our sample group, an EMT of 7 mm could only be achieved in 86% of cases after two rounds of PL infusion. Our findings suggest that excessively thin endometrium, as exhibited by the thinnest case in our study at 4 mm, could be a predictive factor of PRP therapy failure. While PRP may ameliorate thin endometrium to a certain extent, it does not always succeed. Previous literature has reported that PRP can stimulate EMT growth, but responses have been inconsistent, largely due to variations in protocols [21].

Our study also highlights that an increase in uterine injuries decreases the likelihood of successful pregnancy, even when optimal EMT is achieved. Surgical interventions, such as myomectomy, cesarean section, and curettage, could potentially cause dysfunction of the implantation that extends beyond endometrial thickness, as these procedures can impair the endometrial basal layer. Additionally, conditions like chronic endometritis may induce chronic endometrial inflammation, leading to subsequent adhesions [45]. Bu et al. [46] asserted that intrauterine operations primarily contribute to thin endometrium and demonstrated that a thin EMT in conjunction with a normal uterine cavity could result in significantly better LBR. Within our study, PL was ineffective in ameliorating severely damaged endometrium. Thus, future research should focus on defining the surgical threshold and the types of procedures that could better determine the suitable candidates for PRP therapy.

We found a notable increase in the concentration of PDGF-BB in the pregnancy group post-PL infusion. Chang et al. [12] found that PRP could improve pregnancy rates for women with thin endometrium by increasing growth factors, including PDGF-BB, four fold. Our findings suggest that PDGF-BB might be instrumental in enhancing endometrial receptivity beyond mere endometrial thickness. PDGFs, particularly PDGF-BB, the most active isoform in the PDGF family, are prevalent growth factors in PRP, known to activate several signaling pathways and thus facilitate cell migration, proliferation, growth, and tissue regeneration [47,48]. Zhang et al. [49] reported that PDGF-BB not only improved the biological function of menstrual blood-derived stromal cells via the AKT/NF-κB signaling pathway, but also enhanced tissue repair capabilities. Further research is imperative to fully comprehend the role of PDGF-BB in endometrial receptivity.

This study presents several strengths worth noting. First, it exclusively assessed RIF patients with thin endometria, thereby effectively excluding confounding factors such as intrauterine synechiae. Secondly, the analysis was thorough, comprising both a baseline platelet count and the quantification of select growth factors in PL. Unlike other studies, the current research sought to delineate the effects of PL on EMT. PL, an acellular plasma solution derived from disrupted platelet aggregates, contains growth factors, cytokines, and related proteins released upon platelet activation and lysis [50,51]. Using a standardized protocol for PL preparation minimized batch-to-batch variation, thus optimizing the reproducibility of the study [52]. Moreover, the short half-life of growth factors and cytokines necessitated controlling the interval between PL preparation and administration to under 30 min. The use of PL also minimized concerns associated with leukocytes, which are present in many PRP products.

However, this study does have certain limitations. First, our primary endpoint was EMT ≥ 7 mm, with ET only conducted under these conditions. Consequently, our data do not clarify whether PL can enhance endometrial receptivity when EMT is <7 mm. Kim et al. [13] posited that PRP can bolster the IR, CPR, and LBR of patients with thin endometria, even when the endometrium remains thin post-PRP therapy. This novel perspective warrants more extensive molecular research. Secondly, our exclusion criteria may have restricted the study’s external validity, demonstrating the protocol’s applicability in a rather limited population. Lastly, the small sample size might introduce confounding variables, which could potentially skew clinical results. Like any evaluation study design, there are also limitations to the inferences and conclusions we can draw from a pre-test and post-test without a control group.

## 5. Conclusions

This study examined the determinants influencing the effectiveness of intrauterine PRP infusion in cases of RIF associated with a thin EMT. Our findings highlight that diminished EMT can serve as a predictive parameter for the failure of PRP treatment to augment EMT. Furthermore, the study revealed that the number of previous uterine surgical interventions is a significant prognostic marker for predicting unsuccessful pregnancy outcomes subsequent to embryo transfer. These insights should be carefully considered when selecting suitable patients for PRP treatment targeting thin endometrium, thereby enhancing the prospect of successful interventions and improving patient outcomes.

## Figures and Tables

**Table 1:** General patient characteristics.

Patients (n)	77
Maternal age (years)	38.6 ± 6.5
Paternal age (years)	39.4 ± 6.7
BMI (kg/m^2^)	22.3 ± 2.9
Infertility duration (years)	2.8 (1.1–5.0)
Primary infertility (n)	21
Secondary infertility (n)	56
Number of previous failed IVF cycle	3 (2–6)
Number of previous uterine surgery	1.7 ± 1.6
Etiology of infertility (n)	
Male factor	4
Female factor	23
Ovulation factor	20
Tubal factor	3
Mixed (MF + FF)	50
Mean serum platelet count (×10^3^/µL)	289.4 ± 75.2
Growth factor concentrations in PL	
PDGF-BB (pg/mL)	24,380.0 ± 4968
PDGF-AB (pg/mL)	14,977.7 ± 2555.5
VEGF (pg/mL)	1419.7 ± 642.5
VEGF-A (pg/mL)	636.5 ± 292.3
TGF-β1 (pg/mL)	308,800.0 ± 62,160

Note: Age, BMI, and injury times are presented as mean ± SD. Infertility duration and number of failed IVF cycles are presented as median (interquartile range). BMI: body mass index; MF + FF: Male factor + Female factor. Growth factor concentrations in plasma-rich platelet lysate (PL) were determined using Multiskan SkyHigh Microplate Spectrophotometer (Thermo Fisher Scientific Inc.; Waltham, MA, USA). Growth factor concentrations in PL were quantified using enzyme-linked immunosorbent assay kits (ELISA; Abcam). All values are expressed as mean ± SEM.

**Table 2:** Clinical outcomes of intrauterine autologous PL infusion.

Patients (n)	77		
EMT before PL infusion (mm)	6.3 ± 0.9		
EMT after PL infusion (mm)	7.9 ± 1.4		
Mean EMT increase (mm)	1.9 ± 1.7		
	ET group (n = 66) EMT ≥ 7	Cancel ET group (n = 11) EMT < 7	*p*-value
Female age (years)	38.5 ± 6.9	38.8 ± 3.8	0.85
Male age (years)	39.1 ± 7.4	38.9 ± 2.4	0.84
BMI (kg/m^2^)	22.2 ± 2.9	23.6 ± 2.8	0.13
Infertility duration (years)	3.8 ± 3.7	3.5 ± 2.6	0.80
Primary/secondary infertility (%)	(29%/71%)	(18%/82%)	0.41
Gravidity (n)	1.8 ± 1.6	2.8 ± 1.7	0.06
Parity (n)	0.4 ± 0.7	0.4 ± 0.5	0.99
Number of previous failed IVF cycle	4.6 ± 4.0	2.8 ± 1.5	0.011 ^a^
Number of previous uterine surgery	1.55 ± 1.5	2.54 ± 1.9	0.053
Mean serum platelet count (×10^3^/µL)	289.6 ± 76.7	276.8 ± 58.9	0.62
EMT before PL infusion (mm)	6.4 ± 0.7	5.7 ± 1.3	0.02 ^a^
EMT after PL infusion (mm)	8.3 ± 1.1	5.8 ± 0.8	0.000
PI of uterine arteries			
before PL	1.98 ± 0.5	2.2 ± 0.5	0.21
after PL	1.97 ± 0.64	2.2 ± 0.64	0.38
RI of uterine arteries			
before PL	0.79 ± 0.1	0.83 ± 0.04	0.25
after PL	0.80 ± 0.1	0.81 ± 0.1	0.58
Overall outcomes after post-PL ET group			
Number of transferred embryos	1.8 ± 1.0	-	
Implantation rate, % (n)	21% (25/119)	-	
Clinical pregnancy rate, % (n)	33% (22/66)	-	
Miscarriage rate, % (n)	36% (8/22)		
Live birth rate, % (n)	21% (14/66)	-	
Gestational age at delivery (weeks)	37.1 ± 3.8		
Birth weight (g)	2876 ± 694		
Day 3 ET cycle (n = 13)			
Number of transferred embryos	3.2 ± 0.9		
Implantation rate, % (n)	9.5% (4/42)		
Clinical pregnancy rate, % (n)	23% (3/13)		
Miscarriage rate, % (n)	67% (2/3)		
Live birth rate, % (n)	7.7% (1/13)		
Gestational age at delivery (weeks)	39		
Birth weight (g)	2990		
Day 5 ET cycle (n = 53)			
Number of transferred embryos	1.5 ± 0.6		
Implantation rate, % (n)	27% (21/77)		
Clinical pregnancy rate, % (n)	36% (19/53)		
Live birth rate, % (n)	24.5% (13/53)		
Miscarriage rate, % (n)	31.% (6/19)		
Gestational age at delivery (weeks)	36.9 ± 3.9		
Birth weight (g)	2867.9 ± 720		

Data presented as mean ± SD; *p*-value through Mann–Whitney U test; ^a^ *p*-value < 0.05. PL: platelet-rich plasma lysate; ET: embryo transfer; EMT: endometrial thickness; PI: pulsatility index; RI: resistive index.

**Table 3:** Clinical embryo transfer outcomes following intrauterine autologous PL infusion.

Patients (n)	66		
	Pregnant Group (n = 22)	Non-Pregnant Group (n = 44)	*p*-Value
Female age (years)	38.8 ± 7.4	38.1 ± 6.0	0.74
Male age (years)	39.5 ± 7.4	38.4 ± 7.4	0.57
BMI (kg/m^2^)	22.6 ± 3.1	21.4 ± 2.3	0.12
Infertility duration (years)	3.6 ± 3.4	4.2 ± 4.1	0.59
Primary/secondary infertility, %	(29%/71%)	(18%/82%)	0.72
Gravidity (n)	1.8 ± 1.6	2.8 ± 1.7	0.06
Parity (n)	0.4 ± 0.7	0.4 ± 0.5	0.99
Number of previous failed IVF cycle	4.8 ± 4.1	4.1 ± 3.9	0.52
Number of previous uterine surgery	1.1 ± 1.3	2.4 ± 1.5	0.001 ^a^
Mean serum platelet count (×10^3^/µL)	295.8 ± 80	275.9 ± 55.3	0.30
EMT before PL (mm)	6.4 ± 0.66	6.3 ± 0.8	0.48
EMT after PL (mm)	8.8 ± 0.93	8.8 ± 1.73	0.90
Mean ET No. on Day 3 (N = 13)	2.3 ± 1.2	3.5 ± 0.7	0.05
Mean ET No. on Day 5 (N = 53)	1.5 ± 0.61	1.4 ± 0.60	0.52
PI of uterine arteries			
before PL	2.03 ± 0.6	1.87 ± 0.48	0.30
after PL	1.92 ± 0.65	2.1 ± 0.6	0.47
RI of uterine arteries			
before PL	0.80 ± 0.1	0.8 ± 0.1	0.87
after PL	0.79 ± 0.1	0.81 ± 0.1	0.38

Data presented as mean ± SD; *p*-value through Mann–Whitney U test; ^a^ *p*-value < 0.05. PL: platelet-rich plasma lysate; ET: embryo transfer; EMT: endometrial thickness; PI: pulsatility index; RI: resistive index.

**Table 4:** Factors affecting EMT change and pregnancy outcomes following autologous intrauterine PL infusion.

Factors Affecting EMT Change following Autologous Intrauterine PL Infusion				
	Univariate Analysis		Multivariate Analysis	
Variables	OR (95% CI)	*p*-Value	OR (95% CI)	*p*-Value
Female age, years	0.99 (0.90–1.10)	0.90	0.98 (0.75–1.29)	0.88
Male age, years	1.00 (0.69–1.1)	0.92	0.94 (0.74–1.20)	0.62
BMI (kg/m^2^)	0.85 (0.69–1.1)	0.14	0.80 (0.55–1.17)	0.26
Mean serum platelet count (×10^3^/µL)	1.00 (0.99–1.01)	0.61	1.00 (0.99–1.02)	0.53
Infertility duration (years)	1.03 (0.85–1.24)	0.80	1.14 (0.87–1.51)	0.35
Primary/secondary infertility	0.51 (0.1–2.5)	0.50	0.67 (0.07–6.5)	0.73
Number of previous failed IVF cycle	1.2 (0.92–1.59)	0.17	1.11 (0.75–1.63)	0.60
Number of previous uterine surgeries	0.70 (0.48–1.02)	0.06	0.59 (0.29–1.20)	0.15
EMT Before PL (mm)	2.44 (1.2–4.9)	0.03 ^a^	3.16 (1.03–9.67)	0.04 ^a^
PI of uterine arteries				
before PL	0.6 (0.1–3.53)	0.57	0.25 (0.01–8.57)	0.44
after PL	0.92 (0.07–11.6)	0.95	0.94 (0.01–9.2)	0.98
RI of uterine arteries				
before PL	0.3 (0.01–12.0)	0.49	2.68 (0.01–7.8)	0.95
after PL	0.60 (0.2–13)	0.8	2.2 (0.7–2.2)	0.71
**Factors Affecting Pregnancy Outcomes following Autologous Intrauterine PL Infusion**				
	**Univariate Analysis**		**Multivariate Analysis**	
**Variables**	**OR (95% CI)**	***p*** **-Value**	**OR (95% CI)**	***p*** **-Value**
Female age, years	0.99 (0.92–1.06)	0.73	0.99 (0.83–1.18)	0.91
Male age, years	0.98 (0.91–1.05)	0.57	0.98 (0.83–1.18)	0.75
BMI (kg/m^2^)	0.86 (0.70–1.05)	0.13	1.04 (0.79–1.36)	0.80
Platelet count (×10^3^/µL)	0.99 (0.99–1.01)	0.42	1.00 (0.99–1.005)	0.39
Infertility duration (years)	1.04 (0.91–1.19)	0.59	1.12 (0.88–1.44)	0.36
Primary/secondary infertility	2.5 (0.74–8.93)	0.14	0.56 (0.08–3.88)	0.55
Number of failed IVF-ET procedures	0.96 (0.83–1.09)	0.52	0.97(0.78–1.21)	0.78
Number of previous uterine surgeries	1.85 (1.24–2.77)	0.003 ^a^	2.02 (1.12–3.64)	0.02 ^a^
EMT before PL (mm)	0.48 (0.39–1.55)	0.48	0.54 (0.19–1.52)	0.24
EMT after PL (mm)	1.02 (0.68–1.55)	0.89	1.13 (0.67–1.90)	0.65

Factors with significant ORs in the univariate analysis were included in the multivariate analysis; OR: odds ratio; CI: confidence interval; statistical significance when ^a^ *p* < 0.05; BMI: body mass index; IVF: in vitro fertilization; ET: embryo transfer.

## Data Availability

Not applicable.
